# Disorder and Sorption Preferences in a Highly Stable Fluoride-Containing Rare-Earth *fcu*-Type Metal–Organic Framework

**DOI:** 10.1021/acs.chemmater.3c02849

**Published:** 2024-02-08

**Authors:** A. R. Bonity J. Lutton-Gething, Ben F. Spencer, George F. S. Whitehead, Iñigo J. Vitorica-Yrezabal, Daniel Lee, Martin P. Attfield

**Affiliations:** † Department of Chemistry, School of Natural Sciences, The University of Manchester, Oxford Road, Manchester M13 9PL, U.K.; ‡ Department of Materials and National Graphene Institute, The University of Manchester, Oxford Road, Manchester M13 9PL, U.K.; § Photon Science Institute, The University of Manchester, Oxford Road, Manchester M13 9PL, U.K.; ∥ Department of Chemical Engineering, School of Engineering, The University of Manchester, Oxford Road, Manchester M13 9PL, U.K.

## Abstract

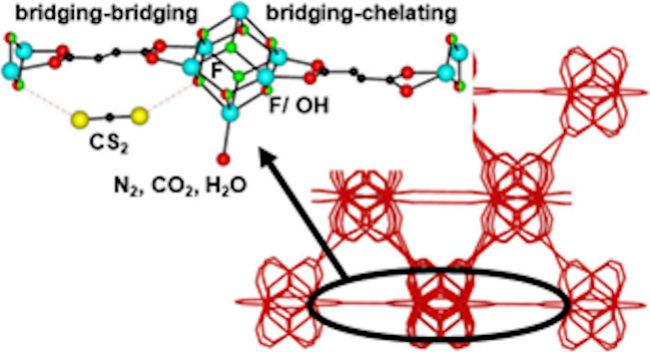

Rare-earth (RE) metal–organic frameworks (MOFs) synthesized in the presence of fluorine-donating modulators or linkers are an important new subset of functional MOFs. However, the exact nature of the RE_*a*_X_*b*_ core of the molecular building block (MBB) of the MOF, where X is a μ_2 or 3_-bridging group, remains unclear. Investigation of one of the archetypal members of this family with the stable *fcu* framework topology, Y-fum-*fcu*-MOF (**1**), using a combination of experimental techniques, including high-field (20 T) solid-state nuclear magnetic resonance spectroscopy, has determined two sources of framework disorder involving the μ_3_-X face-capping group of the MBB and the fumarate (fum) linker. The core of the MBB of **1** is shown to contain a mixture of μ_3_-F^–^ and (OH)^−^ groups with preferential occupation at the crystallographically different face-capping sites that result in different internally lined framework tetrahedral cages. The fum linker is also found to display a disordered arrangement involving bridging– or chelating–bridging bis-bidentate modes over the fum linker positions without influencing the MBB orientation. This linker disorder will, upon activation, result in the creation of Y^3+^ ions with potentially one or two additional uncoordinated sites possessing differing degrees of Lewis acidity. Crystallographically determined host–guest relationships for simple sorbates demonstrate the favored sorption sites for N_2_, CO_2_, and CS_2_ molecules that reflect the chemical nature of both the framework and the sorbate species with the structural partitioning of the μ_3_-groups apparent in determining the favored sorption site of CS_2_. The two types of disorder found within **1** demonstrate the complexity of fluoride-containing RE-MOFs and highlight the possibility to tune this and other frameworks to contain different proportions and segregations of μ_3_-face-capping groups and degrees of linker disorder for specifically tailored applications.

## Introduction

Rare-earth (RE) metal–organic frameworks (MOFs) synthesized in the presence of fluorine-containing modulators or linkers form an important new subset of functional MOFs that display outstanding potential for a host of applications including catalysis, adsorption, separation, sensing, and bioimaging.^1−5^ These MOFs contain complex inorganic molecular building blocks (MBBs) consisting of the RE ion, the coordinating atoms of the multitopic organic linkers, and additional simple terminal or bridging molecules or groups.^6^ Recently, Vizuet et al. recharacterized a Ho-UiO-66 MOF^7^ synthesized in the presence of a fluorine-containing modulator and proposed a RE_6_F_8_ core within the MBB.^8^ They suggested that fluorine-containing modulators or linkers can donate fluorine atoms to developing MBBs during the synthesis so incorporating F^–^ ions into the resultant MOF. They also suggested that members of this previously reported group of MOFs synthesized in the presence of fluorine molecules may also contain a significant fluorine content within the MBB of the MOF. This indicates that there is a certain degree of uncertainty in the chemical composition and structure of the MBBs within these RE-MOFs.

One commonly occurring MBB in the RE-MOFs is the 12-connected hexanuclear RE_6_X_8_R_12_ MBB that contains an octahedron or trigonal antiprism of RE^3+^ ions face-capped by μ_3_-X groups.^7,9−11^ The 12 edges of the RE_6_ polyhedron are bridged by the bidentate groups of the ditopic organic linkers, R, that connect the MBBs to form the framework. Many of the RE-MOFs containing this RE_6_X_8_R_12_ MBB were initially reported as possessing face-capping μ_3_-hydroxy groups.^8^ However, a RE_6_F_8_R_12_ MBB was reported in Ho-UiO-66^8^ and other groups have now reported RE-MOFs containing RE_6_F_*b*_R_12_ (*b* < 8) MBBs but have not proven the chemical identity of the remaining face-capping μ_3_-groups.^12,13^

This 12-connected hexameric RE_6_X_8_R_12_ MBB is the inorganic constituent of the highly stable and important *fcu* topological family of MOFs^14,15^ that are also known to encompass a wide variety of framework disorder.^15−19^ One of the original members of this RE-*fcu*-MOF family is Y-fum-*fcu*-MOF (**1**).^20^ The reported chemical formula of as-synthesized **1** is (DMA)_2_[Y_6_(μ_3_-OH)_8_(fum)_6_(H_2_O)_6_]·(DMF)_5_ (DMA = dimethylammonium, fum = fumarate, DMF = *N*,*N*-dimethylformamide) with each Y^3+^ ion coordinated by nine O atoms from four μ_3_-(OH)^−^ groups, four carboxylate groups, and one terminal H_2_O molecule.^20^ The DMA and DMF were not located in the reported crystal structure, and the MBB of **1** was reported to contain a Y_6_(OH)_8_ core. However, using energy-dispersive X-ray analysis (EDS), Vizuet et al. have shown that **1** contains an unstated amount of fluorine.^8^**1** has also been reported to have an excellent ability to separate hydrocarbon mixtures and to remove hydrogen disulfide and carbon dioxide (CO_2_) from methane.^20−23^ However, there is a dearth of experimental structure–property information concerning the sorption of any guest species within **1** or other members of this family of RE-MOFs synthesized in the presence of fluorine-containing molecules, which is surprising given the current level of interest in their potential applications.^1−5,8^

For these reasons, we reinvestigated the structure of **1** before and during sorption using a variety of complementary techniques to determine the full nature of the framework disorder and the host–guest interactions for a range of relevant linear guest species. In doing so, we have uncovered two sources of disorder within **1** involving the MBB and the fum linker and different favored sorption sites for dinitrogen (N_2_), CO_2_, and carbon disulfide (CS_2_) molecules. The disorder found within **1** demonstrates the complexity of the framework of fluoride-containing RE-MOFs and the possibility to tune this and other frameworks to obtain different proportions and segregations of μ_3_-face-capping groups and degrees of linker disorder for specific applications.

## Experimental Methods

### Reagents

Y(NO_3_)_3_·6H_2_O (99.8%, Sigma-Aldrich), 2-fluorobenzoic acid (97%, Sigma-Aldrich), H_2_fum (99%, Sigma-Aldrich), DMF (99,8%, Alfa Asear), methanol (MeOH, 99.8%, Sigma-Aldrich), and CS_2_ (>99.9%, Sigma-Aldrich) were used as received without further purification. Distilled water was obtained using a Milli-Q system (18 MΩ cm resistivity at 25 °C).

### Synthesis and Activation of **1**

H_2_fum (10.1 mg, 0.087 mmol), Y(NO_3_)_3_·6H_2_O (33.4 mg, 0.087 mmol), 2-fluorobenzoic acid (195 mg, 1.392 mmol), DMF (2.7 mL), and deionized water (0.7 mL) were combined in a 20 mL scintillation.^20^ The vial and contents were sonicated for several minutes to ensure dissolution before being placed in an oven and heated at 115 °C for 72 h. Once cooled to room temperature, the colorless octahedral crystals were collected by filtration, washed with DMF, and air-dried. Attempts to form the fluoride-free framework of **1** by quantitative substitution of 2-fluorobenzoic acid for 2-chlorobenzoic acid failed to yield any Y-fum-*fcu*-MOF product in agreement with other researchers who report the synthesis of other RE-*fcu*-MOFs only in the presence of fluorine-containing modulators or linkers.^24,25^

As-synthesized **1** was solvent-exchanged with MeOH (MeOH-exchanged **1**) by covering as-synthesized **1** crystals with 3 mL of MeOH twice daily for 5 days. These crystals were then activated in situ using a dry N_2(g)_ cryostream or ex situ by heating for 18 h at 80 °C under air (activated **1**).

### Single-Crystal X-ray Diffraction

Single-crystal X-ray diffraction studies were performed on a Rigaku Oxford FR-X diffractometer equipped with a dual X-ray source and a Hypix 6000HE HPC detector. All data were collected using Cu K_α_ (λ = 1.542 Å) or Mo K_α_ (λ = 0.71073) radiation with suitable single crystals mounted on MiTeGen polymer loops and temperature controlled by means of a dry N_2(g)_ cryostream (Oxford Cryostream 800 plus). CrysAlisPro (v42.49) software was used to collect and reduce the data with adsorption corrections applied using empirical methods using symmetry equivalent reflections combined with measurements at different azimuthal angles using the SCALE3 ABSPACK program. All structures were solved using the SHELXT^26^ program that utilizes an intrinsic phasing method on an hklf4 file that contained the reflections from one twin crystal component only and refined using least-squares refinement methods on all *F*^*2*^ values as implemented within SHELXL.^27^ Both SHELXT and SHELXL were operated through the Olex2 (v1.5) interface.^28^ The crystals studied were merohedrally twinned, and the twin law of each crystal was determined using the TwinRotMat program within PLATON^29^ that generated an hklf5 file containing the overlapped reflections from the twinned components and the non-overlapped reflections from the single components. Subsequent refinement was then continued using the reflection data within the hklf5 file.

The fum linker disorder was determined from residual electron density peaks observed directly after refinement of the remainder of the framework with or without application of a solvent mask using the SQUEEZE program^30^ available in PLATON. This solvent mask was subsequently removed after all the fum linker modes were suitably accounted for. The occupancy of the constituent fum linker atoms within a particular linker arrangement was refined against a single free variable, assuming that the sum of the occupancies of the fum linker modes was 1. Geometric restraints were applied to the minor fum linker mode to ensure that a suitable geometry was maintained throughout subsequent structure refinement.

Non-framework species were located by inspection of the residual electron density after full framework determination. Occupancies of non-framework atoms and molecules were refined freely by assigning the constituent atoms to single free variables. Anisotropic or isotropic atomic displacement parameters were refined with suitable restraints or constraints applied to keep them physically reasonable. Hydrogen atoms were placed in calculated positions and refined with idealized geometries and assigned fixed occupancies and isotropic displacement parameters. In the case where non-framework guest species were particularly disordered, the SQUEEZE procedure was applied on the hklf5 file to estimate the number of electrons within the void space.

### In Situ Gas Adsorption Studies

(i) N_2(g)_: A crystal of MeOH-exchanged **1** was activated in situ under a stream of dry N_2_(g) by heating to 500 K before being held at 500 K for 1 h and then cooled to 100 K prior to collection of the diffraction data. (ii) CO_2(g)_: In situ CO_2_ adsorption studies were performed using a homemade gas rig and an adapted Huber goniometer head.^31^ The gas cell was connected to the gas rig through a detachable Swagelok connection, which allows gases to pass or a vacuum to be applied. A MitiGen polymer mount was permanently situated at the top of the goniometer head to which a suitable crystal was glued. A glass capillary was then fitted over the goniometer and gas inlet.

A single crystal of **1** was selected and activated in situ in the gas cell by heating overnight at 353 K under vacuum. Once full activation was confirmed, the gas cell was then loaded with CO_2_. Data was collected at 298 K under CO_2_ pressures of 1, 2.5, 5, and 10 bar. At each step, the gas cell was allowed to equilibrate for 30 min before collection. Data collection, reduction, solution, and refinement were subsequently carried out as described above. The isotropic atomic displacement parameter of the terminal coordinating oxygen atom of the CO_2_ molecule was fixed at a value of 0.1 Å^2^ to avoid occupancy-atomic displacement correlation effects.

### Ex Situ CS_2(l)_ Absorption

Crystals of MeOH-exchanged **1** (2 mg) were heated under static air at 353 K for 18 h followed by further activation at 373 K for 2 h under dry N_2_ before being placed directly into 1.5 mL of CS_2(l)_. Crystals of **1** were selected after soaking in CS_2_ for 3 h and mounted directly under a precooled dry N_2(g)_ stream at 100 K onto the diffractometer. Data was reduced, solved, and refined using the methods described above.

Crystallographic information files CCDC 2306110-2306115 and 2306118-2306119 contain full details for all crystal structures reported.

### Magic Angle Spinning NMR

MAS NMR spectra were collected at the UK High-Field Solid-State NMR Facility using a Bruker 20.0 T (850 MHz ^1^H Larmor frequency) AVANCE NEO spectrometer equipped with a 1.3 mm HXY MAS probe that was used in ^1^H–^19^F/^13^C double-resonance mode. Samples were packed into 1.3 mm o.d. zirconia rotors and sealed with a Vespel cap. Experiments were acquired at ambient temperature using a MAS frequency of 60 kHz. ^1^H- and ^19^F-pulses of 119 and 91 kHz were used, respectively, and echo sequences were employed to reduce interference from the probe background and to avoid receiver dead-time effects. For {^1^H-}^13^C cross-polarization (CP), 1 ms ^13^C spin-locking at 36 kHz was used and an 80–100% ramp was used for Hartman–Hahn ^1^H spin-locking. The 2D ^1^H–^13^C dipolar correlation (HETCOR) MAS NMR spectrum was recorded under the same conditions as for the {^1^H-}^13^C CP, but with a CP contact time of 0.5 ms, and 3968 transients were coadded for each of the 58 complex (States-TPPI) indirect dimension increments. 2D double-quantum (DQ) single-quantum (SQ) ^1^H–^1^H and ^19^F–^19^F dipolar correlation spectra were recorded using one loop of the *S*_2_recoupling sequence for DQ excitation and reconversion,^32,33^ giving a total mixing time of 0.267 ms. Sixteen transients were coadded for each of the 400 complex (States-TPPI) indirect dimension increments for the ^1^H–^1^H spectrum, whereas 64 transients were coadded for 32 complex increments for the ^19^F–^19^F spectrum. Data fitting/deconvolution was performed using the solids lineshape analysis (SOLA) tool of TopSpin 4.0.9.

### XPS

X-ray photoelectron spectroscopy (XPS) was performed using an ESCA2SR spectrometer (ScientaOmicron GmbH) using monochromated Al K_α_ radiation (1486.6 eV, 20 mA emission at 300 W, 1 mm spot size) with a base vacuum pressure of ∼1 × 10^–9^ mbar. Charge neutralization was achieved by using a low-energy electron flood source (FS40A, PreVac). Survey spectra were measured using 200 eV pass energy and core levels with 50 eV pass energy. Binding energy scale calibration was performed using C–C in the C 1s photoelectron peak at 284.8 eV. Analysis and curve fitting were performed using Voigt-approximation peaks using CasaXPS.^34^

### Elemental Analyses

Quantitative elemental analyses were performed by Medac Ltd., UK using ICP-OES for yttrium and Schöniger flask combustion followed by titration for fluorine.

### Powder X-ray Diffraction

Samples were loaded onto a cut silicon sample holder, and diffraction data were collected using a Philips X’pert diffractometer under ambient conditions in the 2θ range of 3.5–40° using Cu K_α_ radiation. All samples prepared were pure phase samples of **1**.

## Results and Discussion

### Structural Disorder within **1**

**Figure 1:**
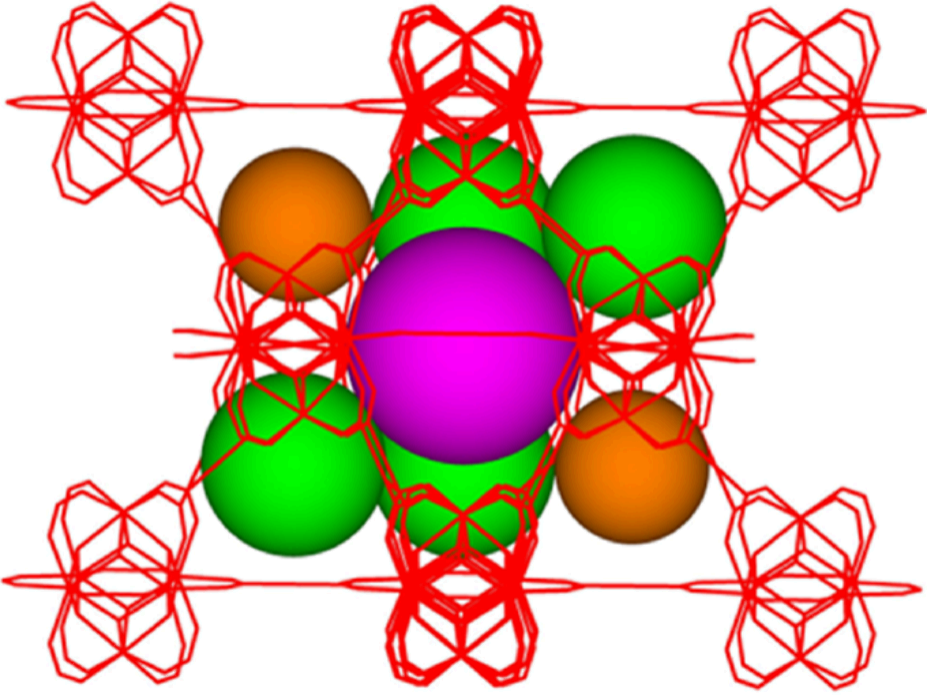
Simplified representation of the framework of Y-fum-*fcu*-MOF (**1**), showing the face-centered cubic arrangement of MBBs containing a series of face-sharing octahedral and tetrahedral cages accessible through triangular windows. Spheres highlight the different cage types, and the size of the spheres in the tetrahedral cages reflects the different sizes of these cages. Color key: All framework atoms and bonds, red sticks; octahedral cages, magenta sphere; tetrahedral cages, green and orange spheres. Two green spheres are omitted for clarity.

The crystal structure of as-synthesized **1** was solved in the cubic *Pn*3̅ space group in which the framework contains one type of octahedral cage and two crystallographically distinct tetrahedral cages of slightly differing void volume, as shown in Figure 1.^35^ The octahedral cage has a diameter of 7.6 Å, and the tetrahedral cages have slightly different diameters of 5.8 and 4.8 Å within the defect-free structure at 100 K as indicated from calculations using PLATON.^36^ Residual electron density in the void volume accounts for the expected solvent DMF, H_2_O, and DMF decomposition product DMA,^37^ the presence of which is validated via solid-state (ss) NMR spectroscopy (*vide infra*).

**Figure 2:**
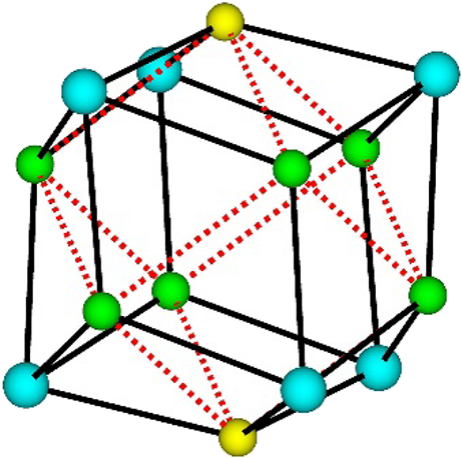
Ball and stick representation of the Y_6_X_8_ core (X = F^−^ or (OH)^−^) of the MBB of **1**. Color key: Y, cyan; X1 (X1 = F1 or (OH)1), green; X2 (X2 = F2 or (OH)2), yellow; YX bonds, black solid lines; X···X non-bonding interactions, red dashed lines.

The crystal structure differs from that previously reported in two significant ways. The first major difference emanates from the structure of the MBB, which refined most stably with a Y_6_(μ_3_-F)_8_ core, as shown in Figure 2, in which the atomic displacement parameters of the face-capping μ_3_-groups of as-synthesized-**1** are the most physically reasonable and consistent with those observed for the other atoms within the framework when refined as F atoms (see Table S1). This suggests that there is a majority of face-capping μ_3_-F sites in the Y_6_(μ_3_-X)_8_ core (X = F^–^ or (OH)^−^) (*vide infra*); however, more precise structural and compositional information concerning the face-capping μ_3_-groups could not be ascertained from the X-ray diffraction data for these adjacent elements in the periodic table.

The presence of fluorine was confirmed in the XPS survey spectrum of activated **1** with the high-resolution spectra of F and Y giving peaks at 684.8 (F), 158.5 (Y 3d_5/2_), and 160.5 (Y 3d_3/2_) eV (see Figure S1) in agreement with the corresponding values for RE-frt-MOF-1 and Y-BCA-3D that contain structurally and chemically similar MBBs^12,25^ suggesting similar chemical states for the F and Y species in these MOFs.

**Figure 3:**
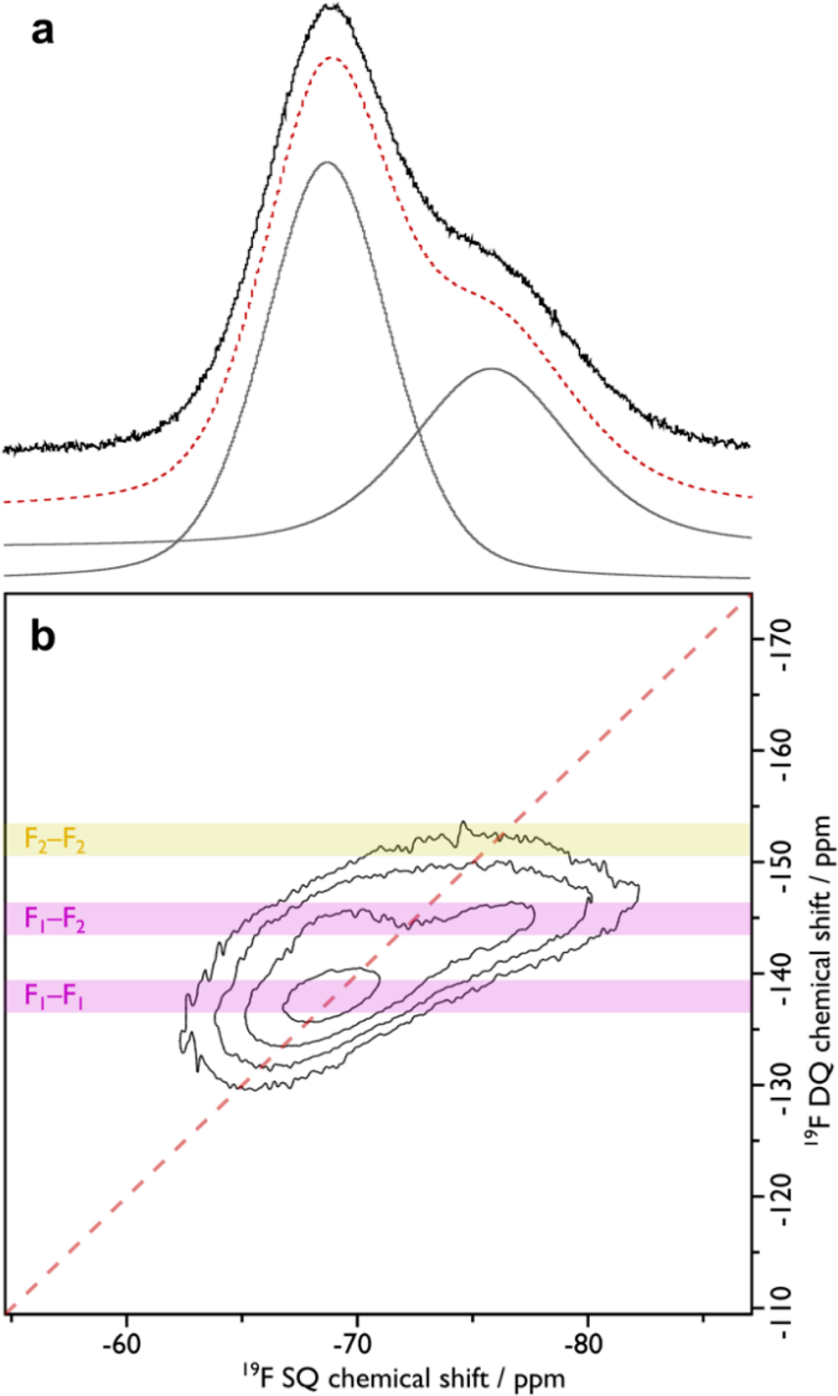
^19^F MAS NMR (a) and ^19^F–^19^F 2D double-quantum single quantum dipolar correlation MAS NMR (b) spectra of MeOH exchanged **1**. The dashed red line in panel (a) is the sum of the peak deconvolution (gray). Guidelines in panel (b) indicate correlations that are present (pink) or absent (yellow).

Elemental analysis of a bulk sample of ex situ activated **1** gave a Y:F ratio of 1:0.88 (Y_6_F_5.3_) differing from an expected value of 1:1.33 for a Y_6_F_8_ core. The presence of fluorine was further confirmed by ssNMR spectroscopy. The ^19^F magic angle spinning (MAS) NMR spectrum of MeOH-exchanged **1**, as shown in Figure 3a, confirmed the presence of two crystallographically independent framework F sites in the Y_6_F_5.3_ core of the MBB (see Figure 2) through two overlapping peaks with chemical shifts of δ{^19^F} = −69 and −76 ppm that were assigned based on their relative intensity to F1 and F2 respectively. These shifts are consistent with μ_3_-F of this MBB type found in another RE-F-*fcu*-MOF, Y-DOBDC MOF^13^ where the fluorine atoms originate from the 2-fluorobenzoic acid modulator used in the synthesis.^8^ A ^19^F–^19^F 2D double-quantum dipolar correlation NMR spectrum (given in Figure 3b) shows strong correlations between the geometrically closer F1/F1 (2.518(3) Å) and F1/F2 (2.520(3) Å) pairs of F atoms in the MBB and minimal correlation for the geometrically more distant F2/F2 (4.331(3) Å) pair. This further confirms the peak assignment and arrangement of fluorine within the MBB. The relative intensity ratio of the −69 and −76 ppm of ^19^F peaks is 1.7:1, which is significantly lower than the expected 3:1 ratio based on the crystallographic multiplicities. This suggests that there is preferential fluorine occupancy of the X2 site compared to the X1 site equating to a Y_6_F1_3.3_F2_2.0_ core and the presence of another type of face-capping μ_3_-group in addition to fluorine in the MBB. The near full occupation of the X2 site by fluorine means that the smaller tetrahedral cage will contain only μ_3_-F^–^ groups within it, while the larger tetrahedral cage will contain ∼55% of the face-capping groups as μ_3_-F^–^ groups, thus modifying the internal chemical nature of the two tetrahedral cages.

**Figure 4:**
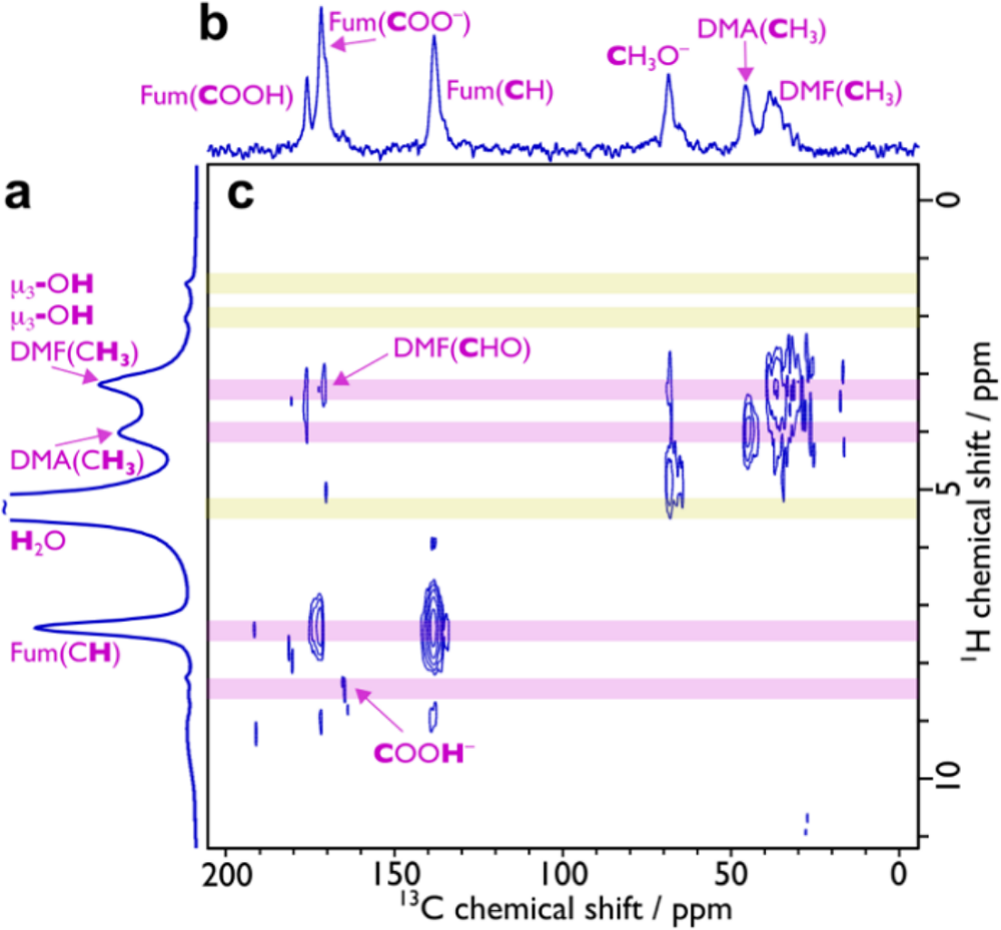
^1^H (a), {^1^H-}^13^C CP (b), and 2D ^1^H–^13^C dipolar correlation (c) MAS NMR spectra of MeOH-exchanged **1**. Guidelines indicate correlations that are present (pink) or absent (yellow).

The presence of a mixture of face-capping μ_3_-groups in this type of MBB has been suggested based on ^19^F MAS NMR spectroscopy results on Y-DOBDC MOF^13^ and EDS measurements on RE-frt-MOF-1 where a range of RE:F ratios were found for different crystals in which there were fewer F atoms than expected for a RE_6_F_8_ core.^12^ The previously reported ex situ IR spectrum for a hydrated sample of **1** shows little clear evidence for the typically most prominent ν(OH) stretch of a μ_3_-(OH)^−^ group in the 3600–3700 cm^–1^ region.^20^ However, the ^1^H MAS NMR spectrum, as shown in Figure 4a and Figure S2, contains ^1^H peaks at δ{^1^H} = 1.5 and 2.1 ppm, which are consistent with the face-capping μ_3_-(OH)^−^ of *fcu*-MOFs.^38,39^ Moreover, these peaks do not exhibit any correlations to ^13^C species in the 2D ^1^H–^13^C dipolar correlation spectrum (Figure 4c), which indicates that they are not related to organic components. A ^1^H–^1^H 2D double quantum dipolar correlation NMR spectrum (given in Figure S3) does not show correlations at these chemical shifts, indicating that the μ_3_-(OH)^−^ environments are discrete. A quantitative analysis of the ^1^H MAS NMR spectrum (see Figure S2 and Table S2) suggests ∼2.4 μ_3_-(OH)^−^ groups per Y_6_ core, which is in reasonable agreement with a value of 2.7 expected from the elemental analysis (Y_6_F_5.3_(OH)_2.7_). The presence of more than one ^1^H peak for μ_3_-(OH)^−^ groups indicates that there are inequivalent OH^–^ environments at occupied X1 sites likely resulting from different ligand binding modes to the MBB near these sites (*vide infra*). Overall, this means that the majority of face-capping μ_3_-groups are μ_3_-F^–^ with a distribution of μ_3_-(OH)^−^ groups that significantly prefer the X1 sites and the larger tetrahedral cage in which they reside.

**Figure 5:**
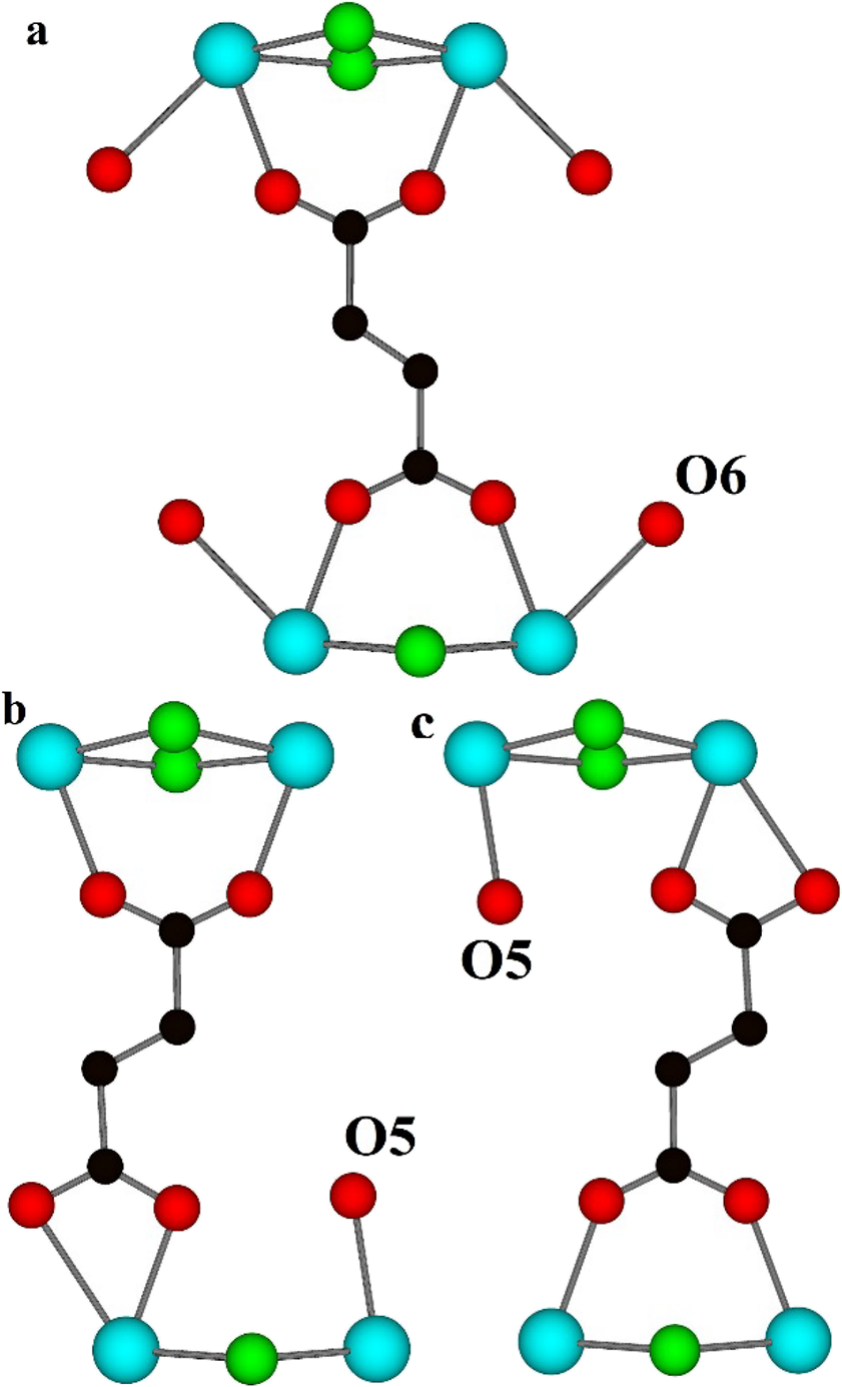
Simplified representation of the bridging–bridging (a) and chelating–bridging (b, c) bis-bidentate modes that a fum linker can adopt between adjacent MBBs. Terminal H_2_O derived from the fum disorder (O5) is shown in panels (b, c), and terminal H_2_O (O6) not derived from the fum disorder is shown in panel (a) but excluded from panels (b, c) for clarity. H atoms have been omitted for clarity. Color key: Y, cyan; F, green; O, red; C, black.

The second major difference from the previously reported structure is that the crystallographically distinct fum linkers are disordered over two possible coordination modes in connecting the Y^3+^ ions in adjacent MBBs, as shown in Figure 5. These coordination modes are the bridging–bridging bis-bidentate mode in which all four O atoms of the fum group bind to four different Y^3+^ ions, as shown in Figure 5a and Figure S4a,^20^ and a chelating–bridging bis-bidentate mode in which both O atoms of one carboxylate group bind to one Y^3+^ ion, and each O atom of the other carboxylate group binds to two different Y^3+^ ions, as shown in Figure 5b,c and Figure S4b,c. The fum linker can switch between the two modes by a simple rotation about the single C–C bond during crystallization of **1**. The different orientations of the fum linker allow them to adopt one of three possible orientations when binding adjacent MBBs, as shown in Figure 5 and Figure S4. The Y–O bond distances in the chelating mode give one longer average distance of 2.63 Å as compared to an average bond distance of 2.32 Å in the bridging mode. The presence of the chelating bidentate coordination at one Y^3+^ ion site means that the adjacent Y^3+^ ion site is coordinatively unsaturated. This additional site is occupied by an O atom located in the Fourier difference electron density maps of what has been assigned a H_2_O (see Figure 5b,c) or placed at the same position as a carboxylate O atom where it has not been resolved (see Figure S4b,c). The interaction of the terminal H_2_O molecules derived from the fum disorder (O5 in Figure 5b,c) with the Y^3+^ ion is stronger than that of the other terminal H_2_O molecule (O6) as reflected by Y–O distances of 2.31(3) and 2.61(3) Å, respectively. The degree of disorder is 25.0(4) % of the fum linkers with 30.0(4) % from one crystallographically independent fum linker and 20.0(4) % from the other.

This organic linker disorder means that the Y^3+^ ions can potentially have one to three coordination sites with terminal water molecules bound potentially yielding highly Lewis acidic sites when **1** is activated to remove coordinating H_2_O molecules. In addition, the triangular windows that contain a fum linker in the chelating–bridging bis-bidentate mode will have a reduced pore size with reduction in H···H intermolecular distances across the pore of between 0.2 and 0.8 Å that may influence molecular sieving aspects of these pores.

The linker disorder was observed in a high proportion of crystals of **1** studied and was found to be maintained in crystals thermally activated at 373 K where the overall crystallinity of **1** was maintained. The linker disorder was not observed for crystals activated to higher temperatures of 500 K where the resolution of the diffraction data and associated crystallinity of **1** had decreased.

This type of chelating–bridging bis-bidentate mode has been reported at 4 of the 12 organic linker sites that are planarly arranged around the MBB in Eu-DOBDC MOF that is a fluoride-containing RE-*fcu*-MOF.^40^ Adjacent MBBs within the Eu-DOBDC MOF are rotated relative to each other to accommodate this coordination mode. However, due to the structure of the fum linker in **1**, this type of linker coordination mode can be accommodated randomly at all linker sites with all MBBs remaining in the same orientation relative to each other unlike that reported for Eu-DOBDC MOF.

The disorder of the organic components was further investigated by ssNMR spectroscopy. The ^13^C MAS NMR spectrum of MeOH-exchanged **1**, given in Figure 4b and Figure S5, provides information on the fum disorder. The peaks associated with the C=C of this linker, centered at δ{^13^C} = 137 ppm, consist of two components, with the major component (∼77%) at δ{^13^C} = 138 ppm and the minor component (∼23%) at δ{^13^C} = 135 ppm (see Figure S5 and Table S3). This infers there are at least two distinct structures for the fumarate linkers, with a degree of disorder consistent with that found from the crystallography. The 2D ^1^H–^13^C dipolar correlation spectrum (shown in Figure 4c) helps to assign the remaining peaks in the ^13^C MAS NMR spectrum. (^13^C, ^1^H) correlations at (172, 7.4) and (173, 7.4) can be attributed to the fum carboxylates that corroborate the conclusions above about the distinct fum linker structures and that at (176, 3.4) can be tentatively assigned to protonated fum linkers. Further evidence for partial linker protonation was observed in the crystallography (*vide supra*), where a longer Y–O bond length was noted for one of the bonds in the chelating binding mode. Moreover, the sum of the integral of the peaks at δ{^13^C} = 172, 173, and 176 ppm is equal to that of the peaks at δ{^13^C} = 135 and 138 ppm. Along with their relative intensities given in Table S3, this suggests that the fum peaks at δ{^13^C} = 173 and 176 ppm are from the chelating–bridging bis-bidentate ligand, with a protonated chelating bidentate fumarate carboxylate group possessing the higher ^13^C chemical shift. The ^13^C MAS NMR spectrum given in Figure 4b contains many more resonances than would be expected for only the fum linker. Resonances could also be expected for DMF, MeOH, and for DMA and formate (HCOO^–^), which can be produced from decomposed DMF during MOF synthesis.^37^ Indeed, (^13^C, ^1^H) correlations are observed at (170, 3.2), (38, 3.2), and (33, 3.2), which can be attributed to DMF, as well as at (46, 4.0) that relate to DMA. The presence of HCOO^–^ species is also detected via correlations at (166, 8.5). Correlations are also observed at (69, 4.8) and (69, 3.3), which can be assigned to Y-bound MeOH/MeO^–^. The (176, 3.4) correlation of the protonated fum linkers to lower-shifted protons (δ{^1^H} = 3.4 ppm) likely indicates that the protonation is at a site where MeOH or DMF is also coordinated.

The ^1^H–^1^H 2D double-quantum dipolar correlation NMR spectrum (given in Figure S3) also highlights close proximities between the DMF and DMA solvent species and between these solvents and fum linker ^1^H, including linker COOH. This shows that these solvent species are framework-bound since isotropic motion in the pores would average out the dipolar coupling. This dynamic averaging is the case for the water molecules in the pores as no correlations are observed.

A quantitative analysis of the ^1^H MAS NMR spectrum (see Figure S2 and Table S2), taking into account the relative intensities in the ^13^C MAS NMR spectrum (see Figure S5 and Table S3), provides a proposed chemical formula for MeOH-exchanged **1** of (DMA^+^)_1.5_[Y_6_(μ_3_-(OH)^–^)_2.4_(μ_3_-F^–^)_5.6_(fum^2–^)_3_(fum^–^)_3_(HCOO^–^)(MeO^–^)_1.5_]·(DMF)_2_·(H_2_O)_29_. This indicates the role of protons in addition to DMA ions to balance the negative charge of the framework and demonstrates the disorder, structural complexity, and a wide variety of possible permutations at the coordination sites in **1**.

### Sorption Behavior of **1**

In situ and ex situ single-crystal X-ray diffraction was used to determine host–guest interactions in **1** for a range of relevant linear guest species to determine structure–property information for possible design and application of **1** or other members of this family of fluoride-containing RE-*fcu*-MOFs.^7,9,10^

**Figure 6:**
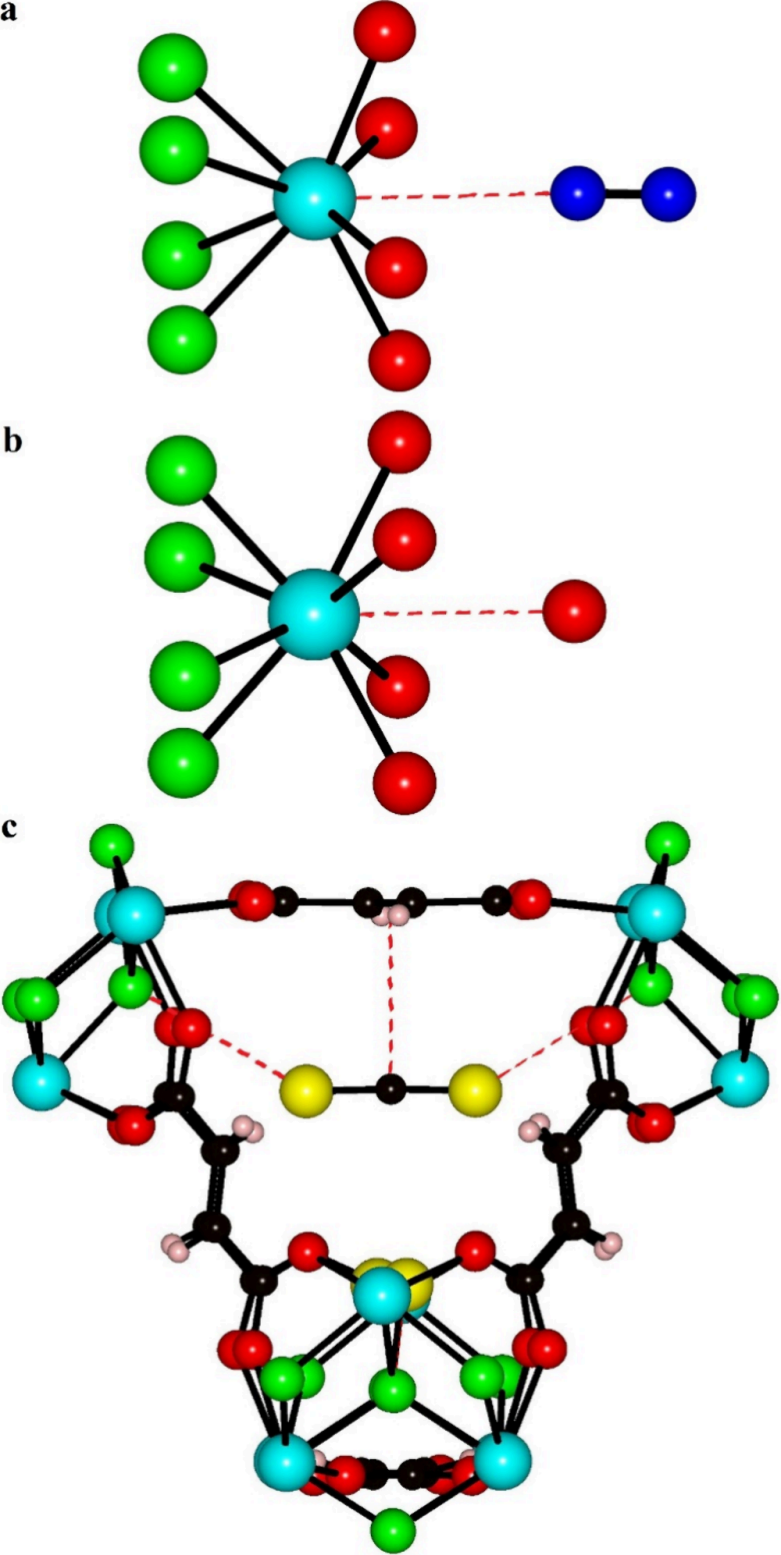
Simplified representations of the location of a N_2_ molecule (a), the end O atom of a CO_2_ molecule (b), and a CS_2_ molecule (c) within **1**. Selected closest contact distances are shown as red dotted lines. Color key: Y, cyan; S, yellow; X (X = F or (OH)), green; O, red; N, blue; C, black; H, pastel magenta.

A crystal of MeOH-exchanged **1** was pretreated as described in the Experimental Methods section. At 100 K and 1 bar of N_2(g)_ (relative pressure, *P*/*P*_o_ of 0.128), N_2_ molecules were located within the octahedral cage of the framework with the lone pair of the N_2_ molecule directed toward the open coordination site of the Y^3+^ ion, as shown in Figure 6a. The rather long Y···N distance of 3.04(1) Å suggests that this coordination is relatively weak and predominantly electrostatic involving the lone pair end of the N_2_ molecule with its associated negative electrostatic potential that will be attracted to the Lewis acidic Y^3+^ ion center.^41^ The freely refined N–N distance of 0.97(2) Å closely matches the expected N–N distance for N_2_ molecules,^42^ and the N_2_ molecules are arranged approximately linearly relative to the Y site (N–N···Y angle 174(3)°). Only 66(2) % of these possible coordination sites are occupied by N_2_. The localization of the N_2_ molecules in this position suggests that it is the site that possesses the strongest interactions with the framework, even though the long Y···N distance indicates that the interaction is weak. Significant residual electron density was also found within the tetrahedral cages, which presumably derives from a combination of disordered additional N_2_ and DMA species.

A crystal of MeOH-exchanged **1** was activated in the gas cell by heating overnight at 353 K under vacuum before being cooled to 298 K as described in the Experimental Methods section. The structure of evacuated **1** revealed a residual electron density of 232 electrons per unit cell after the application of a solvent mask, which is accounted for primarily by the DMA ions. The crystal was then exposed to pressures of 1, 2.5, 5, and 10 bar of CO_2(g)_ at 298 K (see Table 1). A significant increase in residual electron density within the void space was found after the application of a solvent mask above pressures of 1 bar of CO_2_, as shown in Table 1. No clear localization of complete CO_2_ molecules was seen within this residual electron density; however, as the pressure increased, one localized atom position within the void space became evident, and its occupancy increased as the CO_2(g)_ pressure increased (see Table 1). This atom was refined as the terminal O atom of a CO_2_ molecule located within the octahedral cage. The rather long Y···O distance of 2.82(2) Å (10 bar) suggests that this coordination is again relatively weak and predominantly electrostatic involving interaction between the partial negatively charged and electron-rich O atom of CO_2_ with its associated negative electrostatic potential and the partial positively charged and electron-deficient Y^3+^ ion center, as shown in Figure 6b.^43^ The remainder of the CO_2_ molecule is presumably disordered about this terminal O atom within the octahedral cage. Only 45(1) % of these possible coordination sites are occupied by the O atoms of the CO_2_ molecules. The localization of the O atom of the CO_2_ molecules in this position suggests that it is the site with the strongest host–guest interactions, although the long Y···O distance indicates that the interaction is weaker than for the H_2_O molecules found in as-synthesized **1** that have a Y···O distance of 2.60(2) Å. The high degree of CO_2_ disorder and the implied weak host–guest interaction between the framework and CO_2_ molecules agree well with the reported mild regeneration conditions of **1** after CO_2_ adsorption.^44^ The location and occupancy of the CO_2_ molecule do not account for all the residual electron density found, indicating that there are further disordered CO_2_ molecules within the void space in addition to the DMA ions. The favored position of the CO_2_ molecule is different from that found in UiO-66 where it is located within the tetrahedral cages with the end O atom of the CO_2_ molecule interacting with the H atom of the (OH)^−^ group and the rest of the CO_2_ molecule aligning parallel to the aromatic ring.^45^

**Table 1:** Amount of Disordered Electron Density per Unit Cell and the Fractional Occupancy of the Terminal O Atom of a CO_2_ Molecule Found within **1** during an In Situ Single-Crystal X-ray Diffraction CO_2_ Gas Adsorption Experiment^a^

temperature (K), pressure (bar)	relative CO_2_ pressure (*P*/*P*_o_)	disordered electron density per unit cell (e)	fractional occupancy of the terminal O atom of a CO_2_ molecule
298, >0.005	0	232	0
298, 1	0.015	231	0.22(2)
298, 2.5	0.039	572	0.29(2)
298, 5	0.078	630	0.33(2)
298, 10	0.155	736	0.45(1)

a Disordered electron density per unit cell obtained through the application of a solvent mask; fractional occupancy of the terminal O atom of a CO_2_ molecule obtained without application of a solvent mask.

The crystal structure of **1** soaked in CS_2(l)_ collected at 100 K contains one crystallographically distinct CS_2_ molecule located within the larger tetrahedral cage with a 46.0(6) % site occupancy. The CS_2_ molecule lies to one side of the tetrahedral cage center, parallel to a fum linker, as seen in Figure 6c. The distance between the principal axis of the CS_2_ molecule and the plane of the fum linker is 3.29(3) Å, suggesting relatively weak guest–host interactions arising from dispersive contributions. This interaction distance is similar to that found in another CS_2_-MOF system with a parallel alignment of the CS_2_ and the organic linker for which computational studies indicated that the dispersive interactions formed the main contribution to the guest–host interaction.^46,47^ The μ_3_-F···S distance of 3.658(5) Å indicates that any contribution to host–guest interactions from chalcogen bonding is very weak.^43^ Localization of the CS_2_ molecules in the larger tetrahedral cage suggests stronger host–guest interactions in this location, which may result from the preferential ordering of the μ_3_-(OH)^−^ groups in this tetrahedral cavity. It is interesting to note that the most favorable interaction of CS_2_ with the framework is not through the Y^3+^ ion center as it is for N_2_, CO_2_, and H_2_O reflecting the different properties of the molecules. Significant electron density was also identified within the smaller tetrahedral cage, and this could not be assigned to any structurally defined molecular species but is likely to derive from a combination of disordered additional CS_2_ and DMA species.

## Conclusions

This work has fully determined two sources of disorder within the framework of one of the archetypal members of the important family of RE-MOFs synthesized in the presence of fluorine-containing molecules. The MBB of **1** is shown to contain a mixture of face-capping μ_3_-F^–^ and (OH)^−^ groups with a preferential occupation of each group at the different face-capping sites that result in different internally lined tetrahedral cages within the framework. The fum linker is also found to display a disordered arrangement over the possible fum linker positions without an influence on the MBB orientation within the structure. The linker disorder will incorporate Y^3+^ ions with potentially one to two additional uncoordinated sites and differing degrees of Lewis acidity that can be tuned through treatment of **1** under different degrees of activation or with different solvent molecules capping the Y^3+^ sites. Such a disorder may influence the function of this and potentially other fum containing *fcu*-MOFs such as important MOF-801.^35^ Experimentally determined host–guest relationships for simple sorbates demonstrate the favorable sorption sites for different linear molecules that reflect the chemical nature of both the framework and the sorbate species with the structural partitioning of the face-capping species potentially apparent in determining the favored sorption site of CS_2_. The two types of disorder found within **1** demonstrate the complexity of fluoride-containing RE-MOFs and highlight the possibility to tune^48^ this and other frameworks to contain different proportions and segregations of μ_3_-face-capping groups and degrees of linker disorder for specifically tailored applications.

## Supporting Information Available

Tables and figures of additional crystallographic structure information, XPS spectra, NMR spectra, and tables of associated information (PDF)
Crystallographic information files CCDC 2306110-2306115 and 2306118-2306119 (ZIP)
