# Supplementary material for: Disorder and Sorption Preferences in a Highly Stable Fluoride-Containing Rare-Earth fcu-Type Metal–Organic Framework
Source: Chem Mater. 2024 Feb 8;36(4):1957–65. doi: 10.1021/acs.chemmater.3c02849 (PMC10902816; doi:10.1021/acs.chemmater.3c02849)
Supplement: Supplementary file 1 — cm3c02849_si_001.pdf [file cm3c02849_si_001.pdf]

Disorder and sorption preferences in a highly stable fluoride-containing rare earth *fcu*-type metal-organic framework

A. R. Bonity J. Lutton-Gething,[a] Ben F. Spencer,[b,c] George F. S. Whitehead,[a] Iñigo J. Vitorica-Yrezabal,[a†] Daniel Lee,[d] and Martin P. Attfield[a*]

[a]Department of Chemistry, School of Natural Sciences, The University of Manchester, Oxford Road, Manchester, M13 9PL, UK

[b]Department of Materials and National Graphene Institute, The University of Manchester, Oxford Road, Manchester, M13 9PL, UK

[c]Photon Science Institute, The University of Manchester, Oxford Road, Manchester, M13 9PL, UK

[d]Department of Chemical Engineering, School of Engineering, The University of Manchester, Oxford Road, Manchester, M13 9PL, UK

Supplementary Information

Table S1. Comparison of face-capping μ_3-(OH)$^-$ and μ_3-F$^-$ equivalent atomic displacement parameters observed for as-synthesised-**1** at 100 K.

**Atom position (see Fig. 2 for reference)**	**μ_3-O U_{eq} (Å^2)**	**μ_3-F U_{eq} (Å^2)**	**Freely refined occupancy as μ_3-O**	**Freely refined occupancy as μ_3-F**
**μ_3-1**	0.0077(5)	0.0191(5)	1.22(1)	1.04(1)
**μ_3-2**	0.0063(9)	0.0168(9)	1.21(2)	1.00(2)

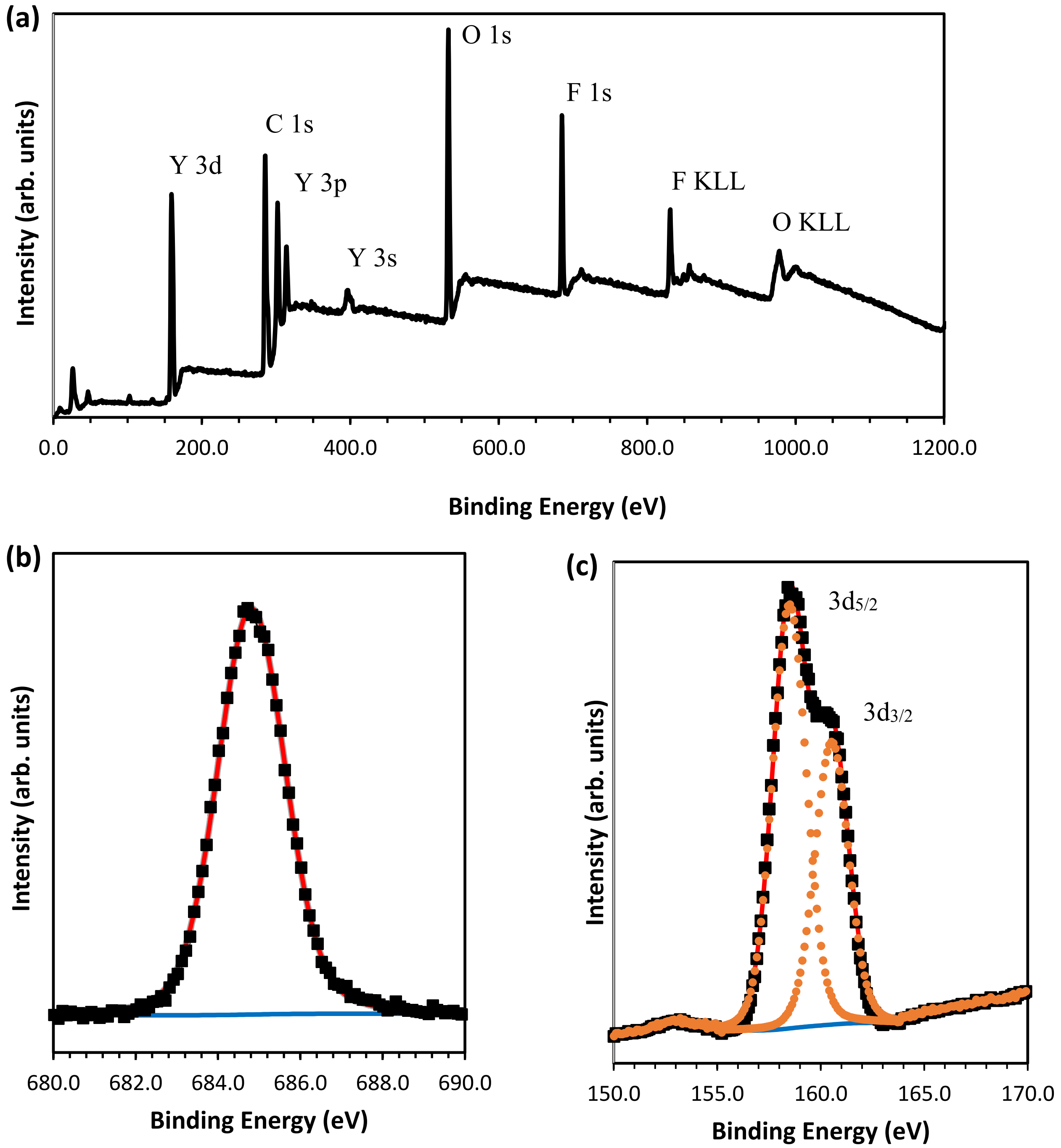

Figure S1. XPS survey (a), F 1s (b) and Y 3d (c) spectra for activated-**1**. Key: black squares or line – observed data, blue line – background, orange circles – contributing fitted peaks, red line – envelope of contributing fitted peaks. The Y 3d peak is fit with one spin-orbit split doublet peak indicating one chemical state, as discussed in the main text.

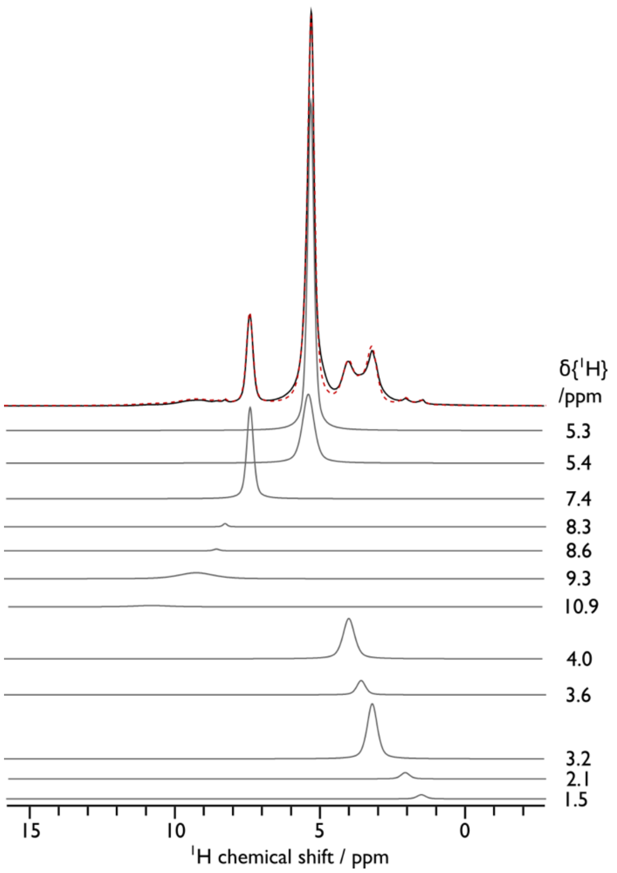

Figure S2. ^{1}H MAS NMR spectrum (top black solid line) of MeOH exchanged-**1** and simulated fit (red dashed line) including the main contributing peaks (below) with their associated chemical shift.

Table S2. Contributions from the deconvolution of the spectrum in Figure S2, their relative integral (to 12 fumerate protons) and their assignment. *Included owing to the difference between simulation and experiment that highlights the need for a contribution at this chemical shift.

^{1}H peak assignment	^{1}H chemical shift / ppm	Relative integral	Linewidth (Hz)
μ_3-OH	1.5	0.7	300
μ_3-OH	2.1	1.1	300
μ_3-OH*	2.4	0.6	800
DMF $(CH_3)_2$	3.2	11	350
MeOH (CH_3)	3.6	2.5	300
DMA $(CH_3)_2$	4.0	9.2	400
DMA (NH_2^+)*	4.9	1.6	350
Pore H_2O	5.3	41.2	220
Pore surface H_2O	5.4	16	500
Fumerate	7.4	12	230
Formate	8.3	0.3	150
DMF (HCO)	8.6	0.2	200
COOH	9.3	4	1200
COOH H-bonded	10.9	0.7	1000

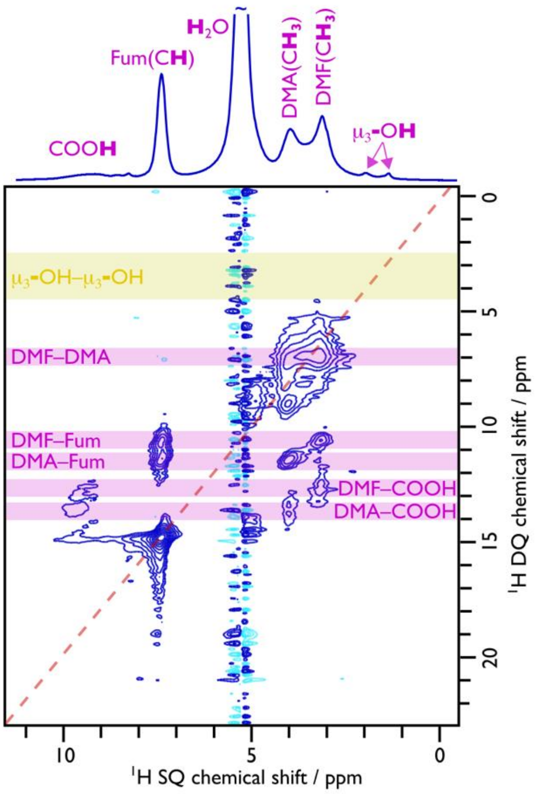

Figure S3. ^1H-^1H 2D double-quantum single-quantum dipolar correlation MAS NMR spectrum of MeOH exchanged-**1**. Guidelines indicate correlations that are present (pink) or absent (yellow).

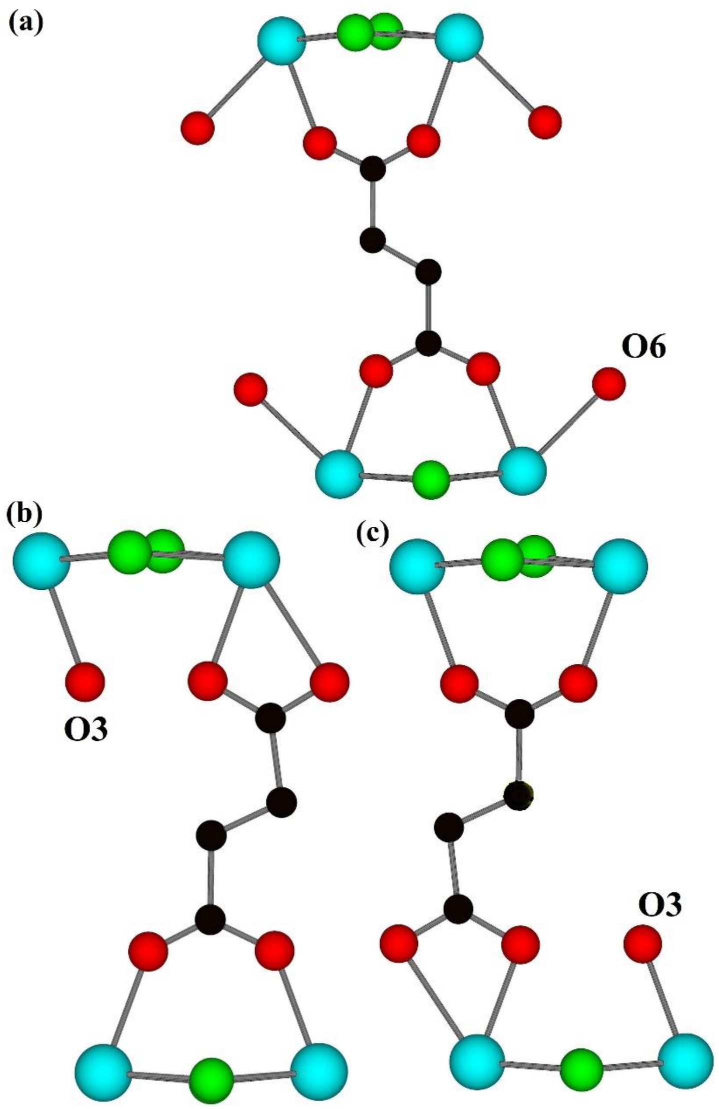

Figure S4. A simplified representation of the bridging-bridging (a) and chelating-bridging (b, c) bis-bidentate modes that a fum linker can adopt between adjacent MBBs. Terminal H_2O derived from the fum disorder (O3) is shown in (b, c) and terminal H_2O (O6) not derived from fum disorder are shown in (a) but excluded from (b, c) for clarity. H atoms are omitted for clarity. Colour key: Y – cyan, F – green, O – red, C – black.

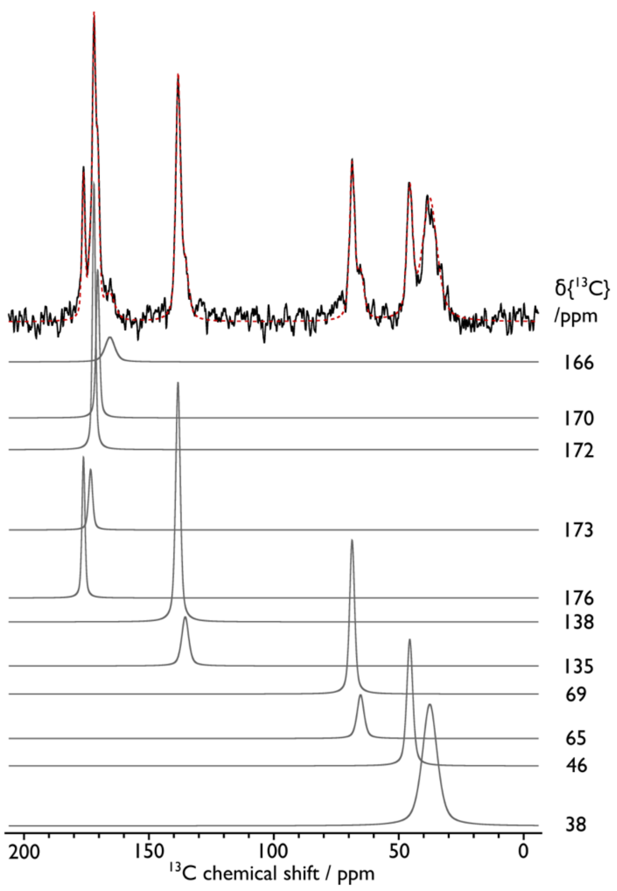

Figure S5. {^{1}H-}^{13}C CPMAS NMR spectrum (top black solid line) of MeOH exchanged-**1** and simulated fit (red dashed line) including the main contributing peaks (below) with their associated chemical shift.

Table S3. Contributions from the deconvolution of the spectrum in Figure SI5, their relative integral (to a total of 12 fumarate CH carbons) and their assignment. Owing to cross-polarization dynamics, the relative integrals will not be fully quantitative for differing ^{13}C environments. *The two discrete methyl ^{13}C resonances of DMF have been approximated by one broader line.

^{13}C peak assignment	^{13}C chemical shift / ppm	Relative integral	Linewidth (Hz)
DMF (CH_3)*	38	14.9	1500
DMA (CH_3)	46	6.2	600
MeO$^–$ ($CH_3O^–$) or MeOH	65	2.5	700
MeO$^–$ ($CH_3O^–$) or MeOH	69	6.3	500
Fum chelate(CH)	135	2.8	700
Fum bridging (CH)	138	9.2	470
Formate ($HCO_2^–$)	166	2.0	1000
DMF (CHO)	170	4.3	350
Fum (bridging COO$^–$)	172	6.5	300
Fum (chelating COO$^–$)	173	2.0	400
Fum (COOH)	176	3.5	300
